# Perspectives of tumor-infiltrating lymphocyte treatment in solid tumors

**DOI:** 10.1186/s12916-021-02006-4

**Published:** 2021-06-11

**Authors:** Shuhang Wang, Jingwei Sun, Kun Chen, Peiwen Ma, Qi Lei, Shujun Xing, Zhongzheng Cao, Shujun Sun, Zicheng Yu, Yarong Liu, Ning Li

**Affiliations:** 1 Clinical Cancer Center, National Cancer Center/National Clinical Research Center for Cancer/Cancer Hospital, Chinese Academy of Medical Sciences and Peking Union Medical College, Beijing, China; 2 Grit Biotechnology Ltd., Shanghai, China; 3 NHC Key Laboratory of Pulmonary Immunological Diseases, Guizhou Provincial People’s Hospital, Guiyang, China; 4 Queen Mary School, Nanchang University, Nanchang, 330006 China; 5 Geneplus-Shenzhen, Shenzhen, China

**Keywords:** Tumor infiltration lymphocyte, Cancer treatment, Immunotherapy, Gene editing, Clinical trials

## Abstract

Tumor-infiltrating lymphocyte (TIL) therapy is a type of adoptive cellular therapy by harvesting infiltrated lymphocytes from tumors, culturing and amplifying them in vitro and then infusing back to treat patients. Its diverse TCR clonality, superior tumor-homing ability, and low off-target toxicity endow TIL therapy unique advantages in treating solid tumors compared with other adoptive cellular therapies. Nevertheless, the successful application of TIL therapy currently is still limited to several types of tumors. Herein in this review, we summarize the fundamental work in the field of TIL therapy and the current landscape and advances of TIL clinical trials worldwide. Moreover, the limitations of the current TIL regimen have been discussed and the opportunities and challenges in the development of next-generation TIL are highlighted. Finally, the future directions of TIL therapy towards a broader clinical application have been proposed.

## Background

### A historical perspective of TIL therapy

TIL therapy is a type of adoptive cellular therapy leveraging the patient’s own immune system to treat tumors. In TIL therapy, TIL is isolated from the tumor site by biopsy or surgery, stimulated and expanded to a large number in vitro with interleukin-2 (IL-2), and then infused back into the patient [1]. In 1982, the pioneer in this field, Dr. Steven Rosenberg, and colleagues at the National Institutes of Health (NIH) isolated TIL from multiple mouse tumor models for the first time [2], and later demonstrated that the combination of cyclophosphamide conditioning, TIL and simultaneous IL-2 administration cured 100% of mice with hepatic metastases and 50% with pulmonary metastases in a MC38 colon adenocarcinoma model [3], laying the foundation for the application of TIL in the treatment of advanced cancers in human. The earliest attempt of TIL therapy in the clinic can be traced back to 1988 and achieved a 60% objective response rate (ORR) in metastatic melanoma [1].

**Fig. 1:**
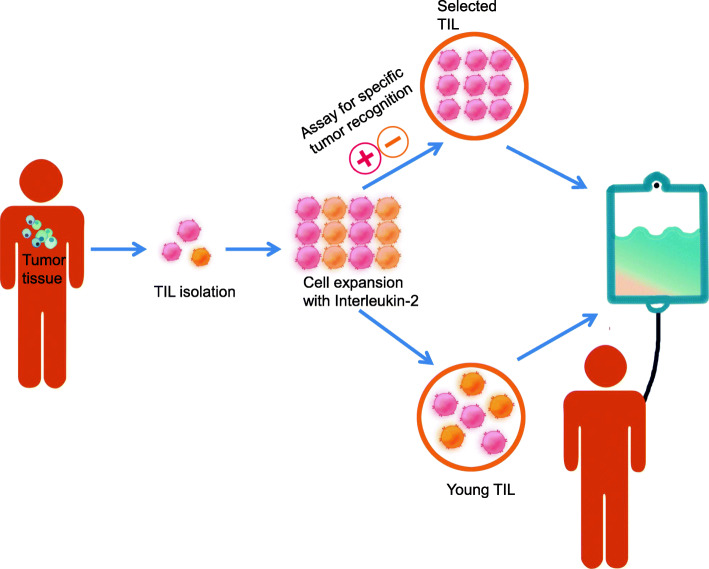
Schematic representation of the production process for TIL therapy. After tumor excision from the patient, tumor is digested into small fragments or a single cell suspension and then expanded in culture with IL-2. In the “selected TIL” approach, expanded cells are selected by their recognition of autologous tumor cells; on the contrary, the “young TIL” approach leaves out this selection step. Then, the TIL culture is expanded to a clinically relevant level and infused back into the patient

The process of generating TIL usually starts from a pre-rapid expansion phase (pre-REP), where TIL is dissociated from or emigrates out of the tumor fragments and goes through preliminary amplification. Then TIL is further expanded in a rapid expansion phase (REP) in response to stimulators, such as IL-2 and/or feeder cells. The traditional procedure of TIL production is assayed for specific tumor recognition and usually takes 6–8 weeks. However, TIL is prone to exhaustion after long-time culturing in vitro and could not persist for long in patients [4, 5]. Besides, the low successful rate of growing autologous tumors in vitro led to a more than 50% dropout rate of patients referred for TIL therapy [6], largely limiting its clinical application [7]. To this end, Dr. Rosenberg and others developed a “Young TIL” approach that rapidly expands TIL for administration without in vitro selection for tumor reactivity [8], which markedly improves the timeliness of TIL production as well as its survival and efficacy in vivo (Fig. 1). Later on, “Young TIL” has shown comparable clinical outcome with traditional TIL in melanoma patients [9, 10]. Besides, the effects of different T cell functional modulators and cytokines on TIL manufacture have also been examined. It has been shown that anti-PD-1, anti-41BB, or anti-CTLA-4 can increase TIL expansion [11, 12]. Combination of IL-2/15/21 can enhance TIL expansion in lung and colorectal cancer and promote CD8+ T cell percentage as well as TCR clone diversity compared to IL-2 alone [13].

Currently, the most widely-used TIL production method is to isolate infiltrating lymphocytes from tumor tissues and then culture and expand these cells in vitro. Previous studies showed that tumor-specific T cells can only be found in the peripheral blood at a minimal level post treatment with PD-1 antibodies, but barely detected before the treatment [14, 15]. Although some researchers isolated PD-1^+^ T cells from the peripheral blood mononuclear cells (PBMCs) and tried to define them as tumor-specific T cells, later studies demonstrated that the percentage of PD-1^+^ T cells in the PBMCs is actually similar between cancer patients and healthy donors, but highly elevated in the lymphocytes infiltrated in tumors [16]. Besides, the PBMCs from both healthy donors and patients contain large numbers of virus-specific T cells [17], further potentiating the rationality of collecting tumor-specific T cells from tumors as the raw material for TIL production.

### Distinguishing features of TIL therapy in solid tumors and the current advances of clinical trials

The following attributes of solid tumor may impose major challenges for developing effective adoptive cellular therapies. Different from hematological malignancies with lineage markers, the high heterogeneity of solid tumors makes it hard to find an ideal target for all tumor cells [18, 19]. Targeting single tumor antigen usually leads to antigen loss or recurrence of more aggressive clones. Moreover, a large percentage of solid tumors is hard to be infiltrated even upon adoptive transfer of a large number of T cells [20, 21]. In addition, it is difficult for T cells to fully exert their function in the tumor microenvironment (TME) due to multiple immune suppressive mechanisms, including but not limited to the upregulation of immune inhibitory molecules, cytokines and metabolites, downregulation of co-stimulatory molecules, and the presence of immune regulatory subsets, such as Tregs, myeloid-derived suppressor cells (MDSCs), and tumor-associated macrophages (TAMs) [22, 23].

TIL may hold some distinguishing advantages for treating solid tumors. Firstly, TIL is composed of T cells with multiple T cell receptor (TCR) clones capable of recognizing an array of tumor antigens, and therefore may be superior in tackling the tumor heterogeneity compared to other adoptive cellular therapies, such as chimeric antigen receptor T (CAR-T) and TCR-T cell therapy. In line with this, TIL has demonstrated better clinical efficacy than CAR-T in solid tumors containing high mutation load, such as melanoma [24, 25]. Secondly, having been stimulated by tumor antigens in *vivo*, TIL tends to dominantly consist of effect memory T (Tem) cells, which express chemokine receptors on the surface, such as CCR5 and CXCR3 [26, 27]. Together with their tumor-specific TCRs, TIL can easily home to antigenically distinct tissues, including tumors, after transferred into patients [28–31]. Last but not least, off-target toxicity has seldom been reported in TIL therapy probably due to the negative selection of TCRs of TIL during the early development of T cell immunity. On the contrary, the engineered tumor-targeting single-chain variable fragments (scFv) in CAR-T or affinity-enhanced TCR in TCR-T product may lead to toxicity if they bear cross-reactivity with antigens on normal tissues [32, 33].

**Fig. 2:**
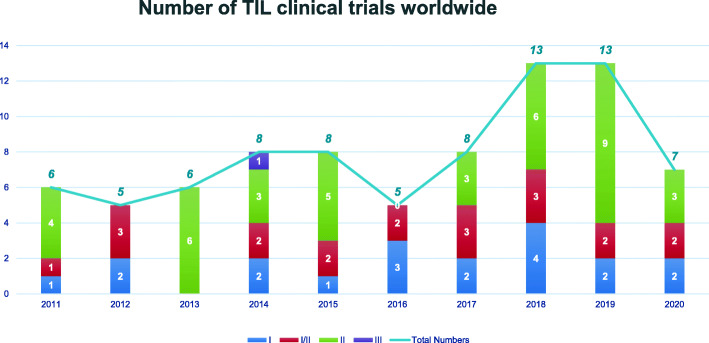
Analysis of numbers of clinical trials on TIL therapy worldwide by year and clinical stage. Data were obtained from PharmaProjects database

**Fig. 3:**
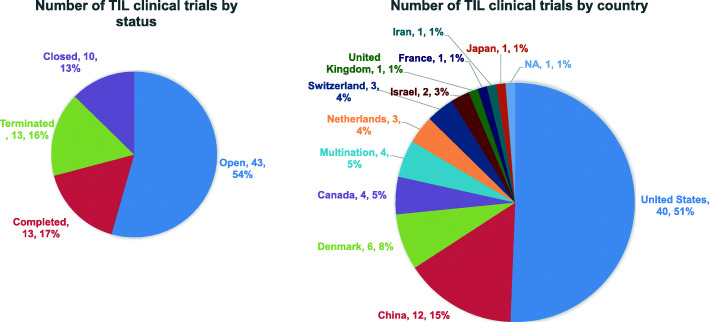
Status and regional disparities in clinical studies of TIL therapies. (Left) The current status of all TIL clinical trials. (Right) Comparison of the geographic localization of TIL clinical trials worldwide. The numbers of trials in each category followed by its percentage among total trials is shown. Data were obtained from PharmaProjects database

**Fig. 4:**
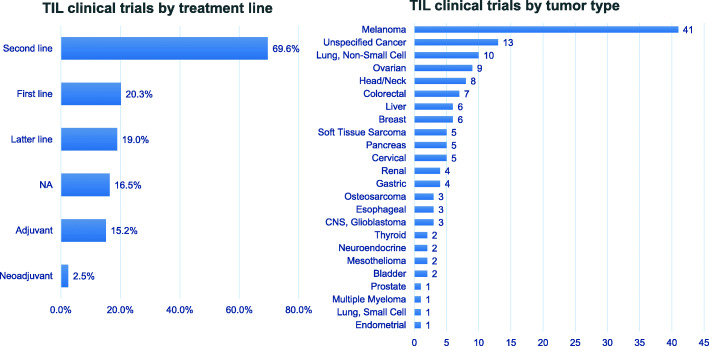
Analysis of TIL clinical trials by treatment line and tumor type. (Left) The percentage of TIL clinical trials applied as different treatment lines. (Right) Number of TIL clinical trials on different tumor types. Data were obtained from PharmaProjects database

In recent years, there have been 79 trials of TIL therapy including 22 kinds of TIL products between 2011 and 2020 (Fig. 2). The trial numbers peaked in 2018 and 2019, along with the success of two phase II trials of TIL products LN144 [34, 35] and LN145 [36] by Iovance in 2018. Currently, 54% (43/79) trials are open, 17% (13/79) have been completed and the rest are either closed or terminated (Fig. 3). Geographic analysis shows that 51% (40/79) of these trials are in the USA and 15% (12/79) are in China (Fig. 3). TIL therapy has mainly been tested as the second treatment line (Fig. 4). Melanoma is still the top tumor type with most clinical trials, followed by non-small cell lung cancer (NSCLC), ovarian cancer, and head and neck cancer (Fig. 4). So far, TIL therapy has shown impressive clinical benefits in metastatic melanoma [37] and advanced cervical cancer [38], even in patients treated with checkpoint inhibitors [39]. Preliminary efficacy has also been demonstrated in NSCLC [40], colorectal cancer (CRC) [41], and breast cancer [42]. The administration of high-dose IL-2 used as standard of care to support the growth and activity of infused TIL [43], however, may restrain the clinical application of TIL therapy. High-dose IL-2 oftentimes induces systemic toxicity that requires intensive monitoring and care [44, 45], and could also promote regulatory T cells that suppress the anti-tumor response of TIL [46]. Although a more than 30% ORR can be achieved in trials with low or intermediate dose of IL-2 [47, 48], most TIL trials still go with high-dose IL-2 infusion. Furthermore, unsustained persistence in vivo and immune suppression in the harsh TME are also obstacles to achieve the maximal outcome of TIL therapy [49].

### Development of next-generation TIL therapy

To reduce toxicities associated with high-dose IL-2 as well as improve the in vivo survival and function of the traditional TIL therapy, next-generation TIL product is under active investigation. Next-generation TIL is genetically modified TIL to either overexpress a gene of interest by viral transduction or knock out (KO) the target gene with technologies like CRISPR or TALEN. However, the development of next-generation TIL may face some major challenges. Gene editing can be technically difficult to achieve in TIL probably due to the varied cellular composition and growth rates of TIL compared with PBMC. Forget et al. has described a method to transduce TIL by retrovirus, leading to transduction efficiency ranging from ~ 31 to ~ 58% in TIL from metastatic melanoma and successful expansion to clinically relevant numbers, but high variability of transduction and expansion rate were observed among patients [50]. Moreover, it is critical but challenging to select the appropriate genes to be targeted in TIL.

So far, the attempts of next-generation TIL in the clinic mainly focused on engineering TIL to overexpress cytokines, such as IL-2 and IL-12. In a phase I/II trial, IL-2 insertion into TIL showed enhanced survival in vitro after IL-2 withdraw but little improvement of persistence in vivo and clinical efficacy [51]. Only 2/12 (17%) ORR was observed in patients who received IL-2 transduced TIL compared to ~ 50% ORR with non-modified TIL along with high-dose IL-2. Another phase I trial using TIL transduced with IL-12 expressed under the control of a nuclear factor of activated T cells (NFAT)-inducible promoter showed favorable clinical efficacy in metastatic melanoma—63% ORR was achieved in patients treated with 0.3−3e^9^ NFAT-IL-12 engineered TILs, which is 10–100-fold less than the cell number used in the traditional TIL therapy [52]. However, unexpected high level of IL-12 and IFN-γ in the serum together with significant toxicity was also observed. On the other hand, the feasibility and functionality of genetically KO of T cell negative regulators in TIL, such as PD-1 [53] and CISH [54], is also being actively tested at pre-clinical stage. How to rationally select or identify the target genes conveying the most benefits for TIL therapy still remains to be explored. In this regard, screening using CRISPR technologies [55, 56], including CRISPR KO, interference (CRISPRi) or activation (CRISPRa), may be promising strategies to identify novel targets for TIL. Moreover, comprehensive safety and function evaluation of genetically modified TIL is required before moving into the clinic. In addition to in vitro characterization, animal models remain to be established to better understand the persistence, function as well as toxicity of modified TIL in vivo.

## Conclusions

Cancer immunotherapy represented by checkpoint inhibitors, especially anti-PD-1/PD-L1 antibodies, and adoptive cellular therapies, such as CAR-T and TCR-T, has revolutionized the field of cancer treatment and significantly prolonged the survival of patients with advanced cancers [57, 58]. However, the response rate is still limited in most cancer types, especially solid tumors [59], which calls for the development of alternative therapeutic strategies. TIL holds some unique advantages in tackling solid tumors, but the laborious, expensive, and time-consuming tissue collection and production process makes TIL only be developed at some leading research institutions and companies in a few countries. There is still considerable room for further improvement and a broader application of this therapeutic approach, for instance, how to establish a standardized and stable production process of TIL from different patients, how to tailor the process based on the features of different tumor types, how to enrich tumor-specific TIL without largely increasing the process time, and how to select prognosis markers to identify patients that potentially benefit the most.

The development of gene-modified TIL holds promise to enhance the clinical outcome of TIL therapy across more indications as well as to enhance safety by decreasing the dependence on high-dose IL-2 infusion and lymphodepletion. Nevertheless, target selection and comprehensive functional evaluation could be critical steps for a successful next-generation product. On the other hand, the combination of TIL therapy with anti-PD-1/PD-L1 antibodies that blockade the immune suppression mediated by the PD pathway at the tumor site showed preliminary favorable outcomes in some recent trials [40, 60]. Other combinational approaches, such as TIL therapy combined with oncolytic viruses, are also under exploration. Similar to CAR-T therapies that show excellent responses in the second- or the third-line setting, there is considerable interest in moving TIL therapy to first-line treatment. The blossom of novel drug investigation at the neoadjuvant and adjuvant stage of treatment also brings opportunity to TIL. Although the development of TIL therapy is currently still in its infancy in China, the advent of CAR-T therapy has helped build a good macro-environment for cell therapy and gene editing industry. The collaboration and joint efforts from research institutions, biotech companies, hospitals as well as the government would drive this field ahead and facilitate the application of TIL therapy to a broader population of patients with solid tumors.

## Data Availability

Not applicable (review).
